# 

**Published:** 1916-05

**Authors:** 


### Publishers' Special Announcements

At this season of the year the Institutions of Dental Learning are bringing forth the 1916 Graduating Classes, the product of three years of careful training. The Seniors will become graduates and pass on into the practice of the Dental Profession and take up their work as a life occupation. The incidents of the class room and operative clinic will pass behind into a dreamy mist of memory and the responsibilities of a modern practice will take their places.

The Dental Register is a favorite with the college students as well as with the practicing dentists and naturally is looked to by both for information on dental subjects as well as to keep posted on the latest wrinkles in dental manufacture which the leading houses are bringing out.

Complete files of the Dental Register are kept by the principal libraries throughout the United States and it is preferentially chosen by conservative readers.

With this issue of the Dental Register the publishers wish to extend their congratulations to all the graduates, that they are entering into the domain of real practice and to express the hope that they will have successful careers, bringing added and accumulative renown to themselves and to the grand institutions from which they are about to come forth.

### ANNOUNCEMENTS

NATIONAL ASSOCIATION OF DENTAL FACULTIES will hold its next meeting at the Seelbach Hotel in Louisville, Ky., July 22-24, 1916.

Kansas City. CHARLES C. ALLEN, *Secy.*

TENNESSEE BOARD OF DENTAL EXAMINERS will hold its next meeting in Nashville, beginning Monday, June 12, 1916, at 10 a. m., and continuing through Friday, June 16th. Information and application blanks may be had by writing to

WALTER G. HUTCHINSON, *Secy.*

308 Eve Bldg., Nashville, Tenn.

NEBRASKA STATE EXAMINERS will meet in Omaha, June 20 to 24, 1916. Full information may be had by addressing DR. J. H. WALLACE, *Secy.*

Omaha, Nebraska.

NORTHERN OHIO DENTAL ASSOCIATION will hold its annual meeting in Cleveland, June 1, 2 and 3, 1916, at the Hotel Statler. Program of the meeting may be had by writing to DR. G. D. LOVETT,

630 Rose Bldg., Cleveland, Ohio.

OFFICERS OF MICHIGAN STATE DENTAL SOCIETY. At the annual meeting held in Detroit, April 13th, the following officers were elected: President, Dr. G. C. Bowles, of Detroit; Vice President, Dr. O. W. White, of Detroit; Executive Council, Dr. C. J. Lyons, of Ann Arbor, and Dr. W. L. Crego, of Saginaw; Secretary, Dr. C. G. Bates, of Durand; Treasurer, Dr. E. J. Chamberlain, of Grand Rapids. The meeting was a great success from every standpoint. Dr. F. B. Morehead, of Chicago, gave a very instructive address on diseases of the mouth and their systemic complications. Dr. W. E. Cummer, of Toronto, Canada, gave a most interesting paper and clinic on method of attaching partial plates and removable bridges, and other valuable plate prosthodontic procedures. Dr. Karl Knoche, of Chicago, presented a most interesting paper and clinic on methods of attaching partial dentures and bridges. Dr. T. P. Hinman presented a moving picture illustration of his technique for manipulating amalgam as a filling material. It was very interesting and vividly demonstrated to the large audience his ideas in a shorter time than would be possible in a paper, and more satisfactorily than could be shown by a chair clinic.

